# Supplementary material for: Molecular recognition of formylpeptides and diverse agonists by the formylpeptide receptors FPR1 and FPR2
Source: Nat Commun. 2022 Feb 25;13:1054. doi: 10.1038/s41467-022-28586-0 (PMC8881469; doi:10.1038/s41467-022-28586-0)
Supplement: Supplementary file 1 — Supplementary Information [file 41467_2022_28586_MOESM1_ESM.pdf]

Supplementary Information

Molecular recognition of formylpeptides and diverse agonists by the formylpeptide receptors FPR1 and FPR2

Youwen Zhuang[1,4], Lei Wang[2,4], Jia Guo[1,3], Dapeng Sun[2], Yue Wang[1,3], Weiyi Liu[1,3], H. Eric Xu[1,3*], Cheng Zhang[2*]

[1]The CAS Key Laboratory of Receptor Research, Shanghai Institute of Materia Medica, Chinese Academy of Sciences, Shanghai 201203, China.

[2]Department of Pharmacology and Chemical Biology, University of Pittsburgh School of Medicine, University of Pittsburgh, Pittsburgh, PA 15261, USA

[3]University of Chinese Academy of Sciences, Beijing 100049, China.

[4]These authors contribute equally to this paper.

[*]Correspondence: H.E.X (Eric.Xu@simm.ac.cn), C.Z. (chengzh@pitt.edu).

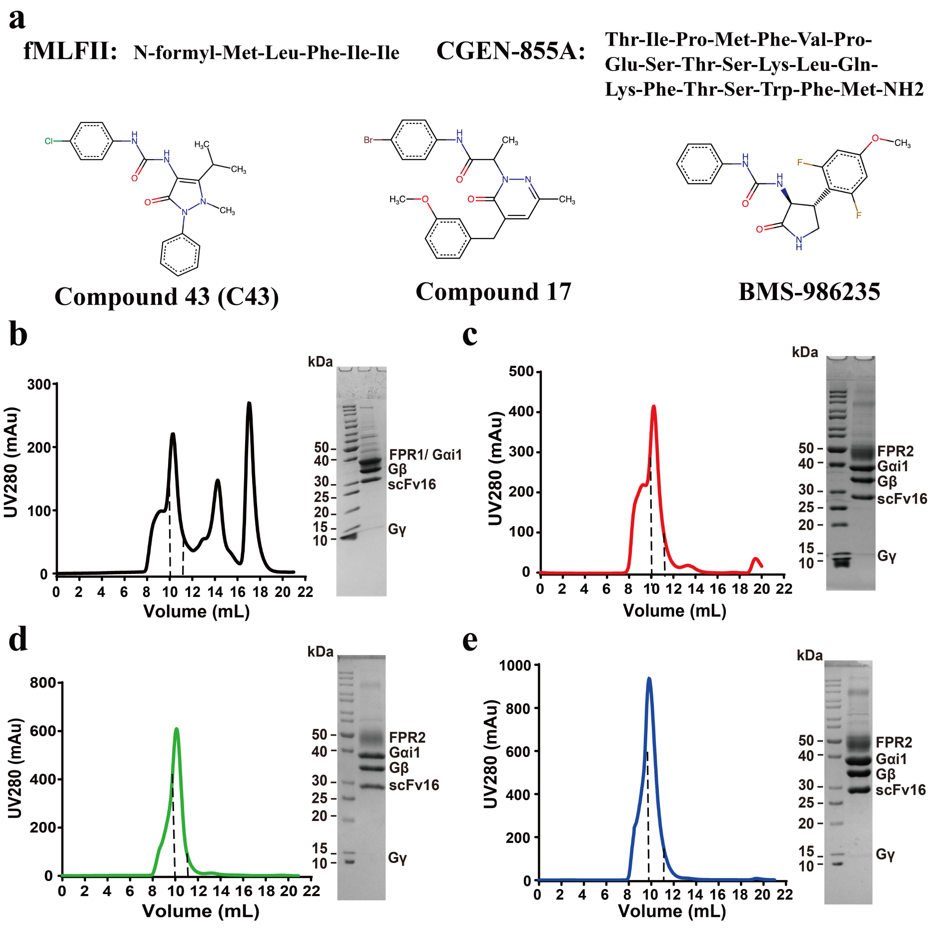

Supplementary Figure 1. Representative FPR agonists and purification of FPR signaling complexes. (a) Chemical structures of peptide agonists fMLFII and CGEN-855A and non-peptide synthetic agonists Compound 43, Compound 17 and BMS-986235. **(b)** Size-exclusion chromatography (SEC) profile and SDS-PAGE image of the fMLFII-FPR1-G_i-scFv16 complex. **(c)-(e)** Size-exclusion chromatography (SEC) profiles and SDS-PAGE images of fMLFII-FPR2-G_i-scFv16, CGEN-855A-FPR2-G_i-scFv16 and C43-FPR2-G_i-scFv16 complexes, respectively. Each panel in b-e shows the result of one-time experiment.

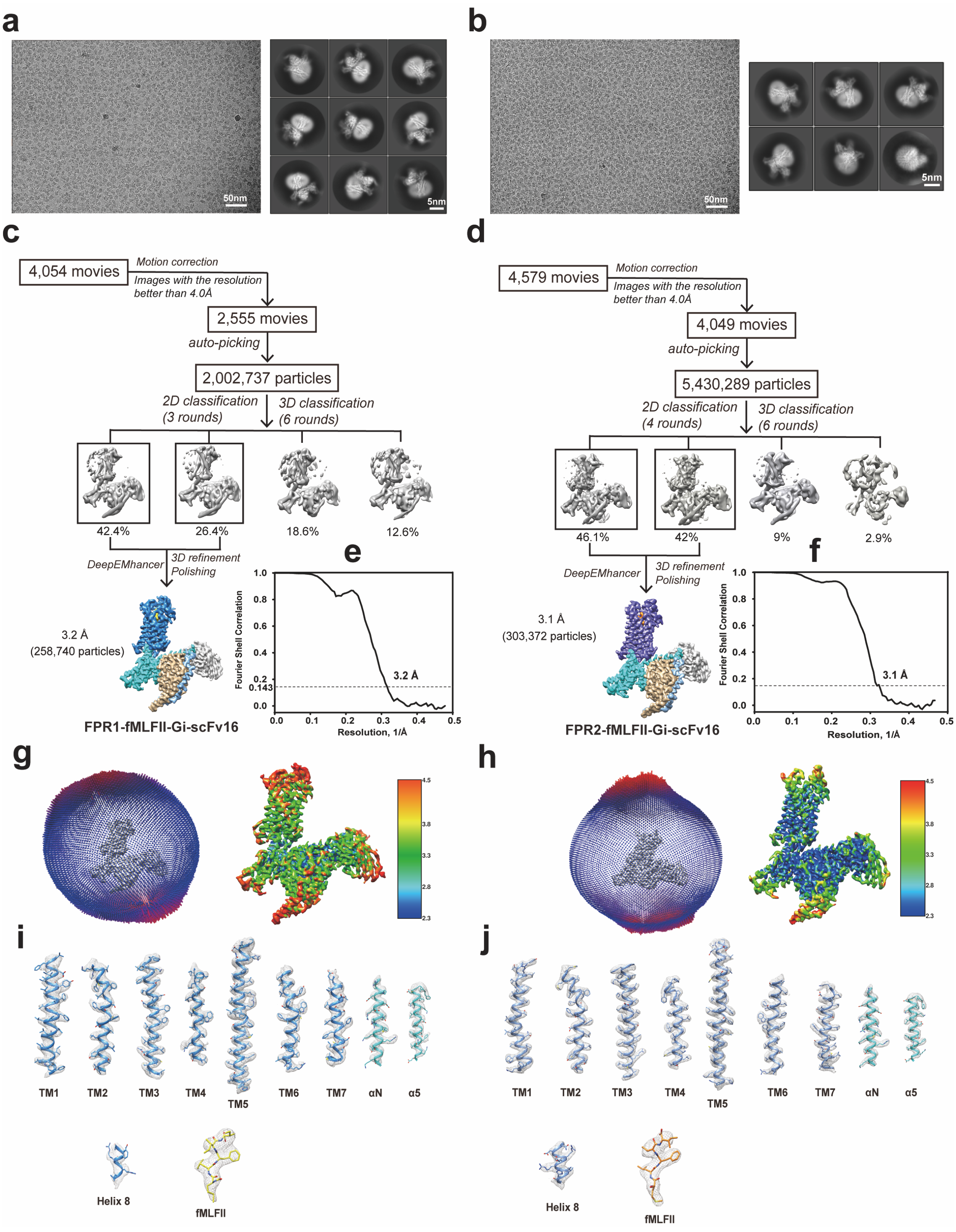

Supplementary Figure 2. Structure determination of the fMLFII-FPR1-G$_i$ and fMLFII-FPR2-G$_i$ complexes. **(a)** and **(b)** Left panel shows one representative cryo-EM micrograph.

Right panel shows representative 2D classification averages of fMLFII-FPR1-G_i **(a)** and fMLFII-FPR2-G_i **(b)**. The 2D averages display structural features of different views. **(c) - (f)** Cryo-EM data processing flowcharts of fMLFII-FPR1-G_i **(c)** and fMLFII-FPR2-G_i **(d)** by Relion 3.1, the final processed global density maps and the 'Gold-standard' Fourier shell correlation (FSC) curves of fMLFII-FPR1-G_i **(e)** and fMLFII-FPR1-G_i **(f)** were also shown, respectively. The global resolution estimated at the FSC=0.143 is 3.2 Å for fMLFII-FPR1-G_i and 3.1 Å for fMLFII-FPR2-G_i. (**g**) **- (h)** Density maps of fMLFII-FPR1-G_i **(g)** and fMLFII-FPR2-G_i **(h)** colored by local resolution (Å), together with the angle distribution maps of particle orientations. The density maps are shown at thresholds of 0.315 and 0.33 for fMLFII-FPR1-G_i and fMLFII-FPR2-G_i complexes, individually. (**i**) Density maps of transmembrane helixes (TM) TM1-TM7 and helix 8 of FPR1, αN and α5 regions of $G_{\alpha i1}$, and ligand fMLFII in fMLFII-FPR1-G_i structure, the density maps were shown at 0.19 threshold. (**j**) Density maps of transmembrane helixes TM1-TM7 and helix 8 of FPR2, αN and α5 regions of $G_{\alpha i1}$, and ligand fMLFII in fMLFII-FPR2-G_i structure, the density maps were shown at 0.2 threshold.

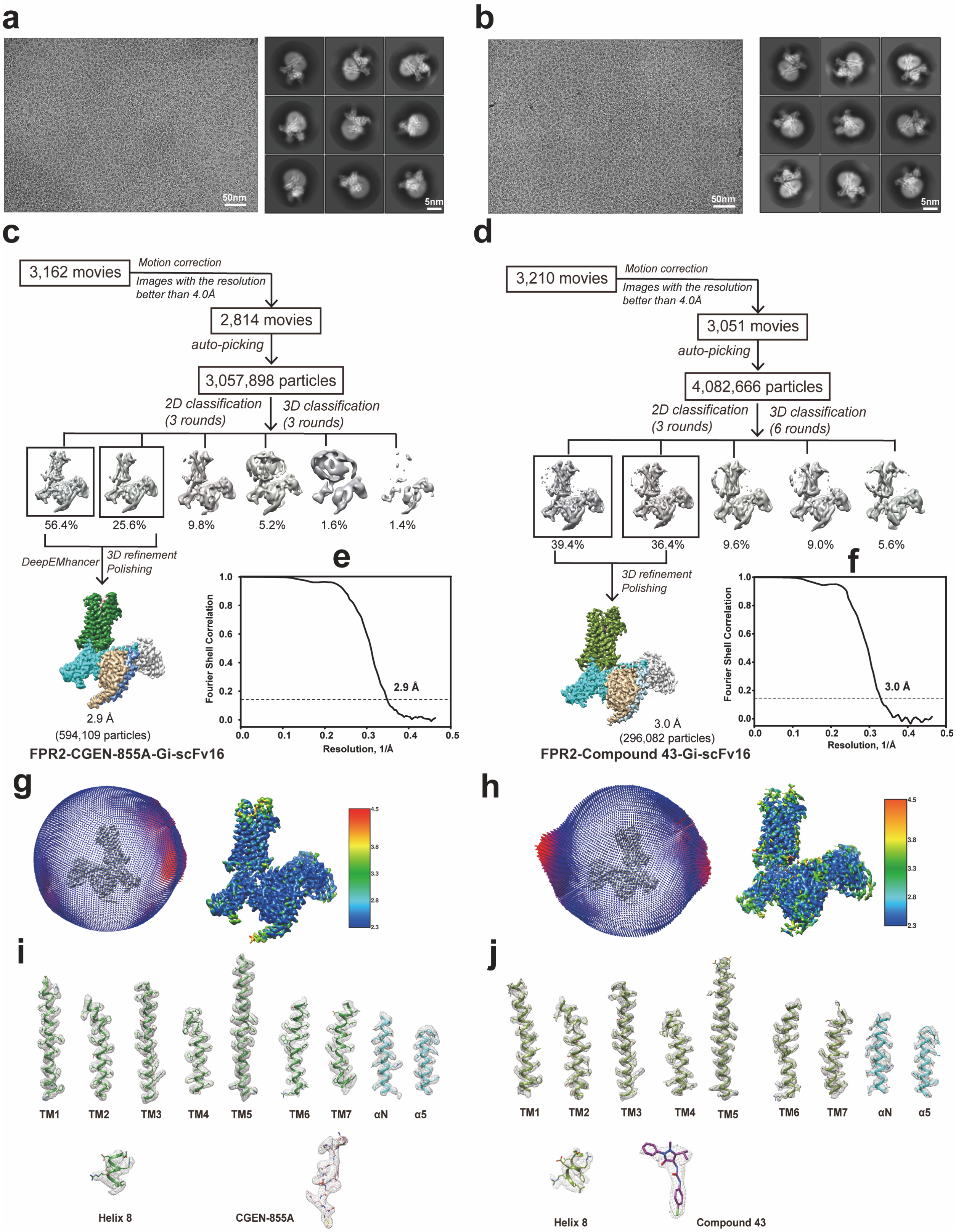

Supplementary Figure 3. Structure determination of the FPR2-G_i complexes with CGEN-855A and Compound 43. (a) - (b) Left panel shows one representative cryo-EM micrograph.

Right panel shows representative 2D classification averages of FPR2-G_i bound with CGEN-855A **(a)** and Compound 43 **(b)**. The 2D averages display structural features of different views. **(c) - (f)** Cryo-EM data processing flowcharts of CGEN-855A-FPR2-G_i **(c)** and Compound 43-FPR2-G_i **(d)** by Relion 3.1, the final processed global density maps and the 'Gold-standard' Fourier shell correlation (FSC) curves of FPR2-G_i complexed with CGEN-855A**(e)** and Compound 43**(f)** were also shown, respectively. The global resolution estimated at the FSC=0.143 is 2.9 Å for CGEN-855A-FPR2-G_i and 3.0 Å for Compound 43-FPR2-G_i. (**g) - (h)** Density maps of FPR2-G_i bound with CGEN-855A **(g)** and Compound 43 **(h)** colored by local resolution (Å), together with the angle distribution maps of particle orientations. The density maps are shown at thresholds of 0.096 and 0.035 for CGEN-855A bound- and Compound 43 bound- FPR2-G_i, respectively. **(i) - (j)** Density maps of transmembrane helixes TM1-TM7 and helix 8 of FPR2, αN and α5 regions of $G_{\alpha i1}$, and ligands in CGEN-855A-FPR2-G_i structure **(i)** and Compound 43-FPR2-G_i structure **(j)**. The density maps were shown at 0.027 and 0.03 threshold for CGEN-855A and Compound 43 bound- FPR2 structures, respectively.

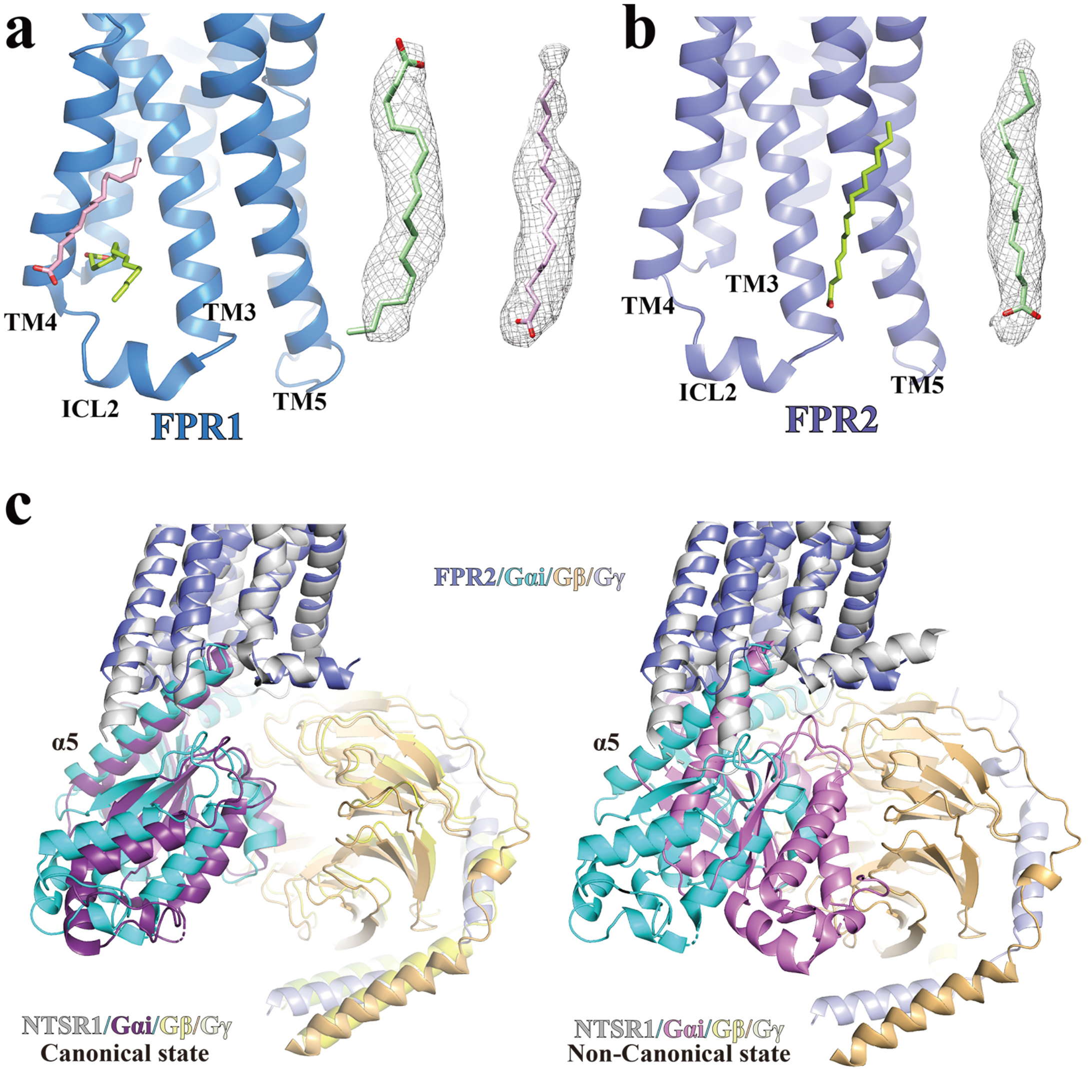

Supplementary Figure 4. Lipids modeled in FPR1 and FPR2 structures and structural comparison of Gi-coupled FPR1, FPR2, and neurotensin receptor 1 (NTSR1). (a) Two palmitic acid molecules modeled in the structure of fMLFII-bound FPR1 and their density maps. **(b)** One palmitic acid molecule modeled in the structure of fMLFII-bound FPR2 and its density map. In both structures, the palmitic acid molecules are bound in a groove above ICL2 formed by TM3, TM4 and TM5. **(c)** Comparison of the FPR2-G_i complex in our structures and the NTSR-G_i complex at the canonical (PDB ID 6OS9, http://doi.org/10.2210/pdb6os9/pdb) and non-canonical state (PDB ID 6OSA, http://doi.org/10.2210/pdb6osa/pdb) based on the alignment of the receptors. The FPR2-G_i complex in our structure is more similar to the NTSR-G_i complex at the canonical state than that at the non-canonical state. ICL: intracellular loop.

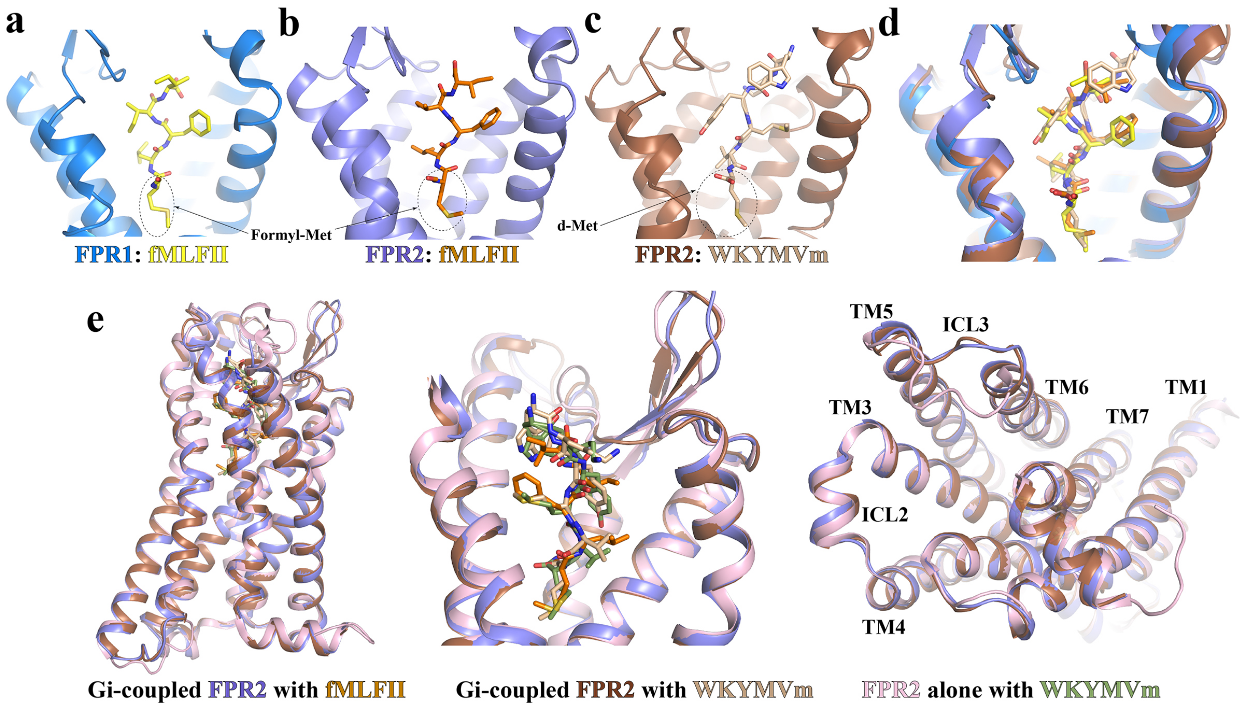

Supplementary Figure 5. Binding poses of fMLFII and WKYMVm. (a) and **(b)** N-terminus-inside binding pose of fMLFII in FPR1 (blue) and FPR2 (slate), respectively. The ligand in FPR2 and FPR1 is colored orange and yellow, respectively. The N-formyl Met is circled. **(c)** C-terminus-inside binding pose of WKYMVm (wheat) in the structure of WKYMVm-FPR2-G_i (PDB ID 6OMM, http://doi.org/10.2210/pdb6omm/pdb). FPR2 in this structure is colored brown. The C-terminal d-Met is circled. **(d)** Alignment of fMLFII and WKYMVm in the three structures shown in a-c. **(e)** Alignment of G_i-coupled FPR2 (slate) with fMLFII (orange), G_i-coupled FPR2 (brown) with WKYMVm (wheat), and FPR2 alone (light pink) with WKYMVm (light green) (PDB ID 6LW5, http://doi.org/10.2210/pdb6lw5/pdb). The left panel shows the overall structural alignment. The middle panel shows the alignment of the peptide ligands. The right panel shows the alignment of the cytoplasmic regions.

a

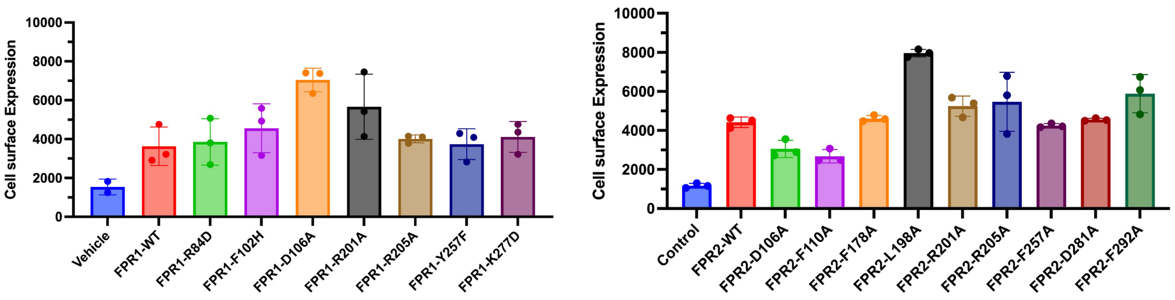

b

FPR1	pEC_{50} (fMLF)
WT	8.02±0.25
F84D	7.25±0.21
F102H	7.71±0.42
D106A	ND
R201A	6.00±0.22
R205A	5.27±0.38
Y257F	6.82±0.59
K277D	7.74±0.50

FPR2	pEC50(CGEN)
WT	6.61±0.57
D106A	NA
F110A	5.41±1.27
F178A	6.49±0.54
L198A	6.30±0.29
R201A	7.29±1.03
R205A	6.85±0.47
F257A	6.42±0.48
D281A	6.83±0.69
F292A	6.28±0.39

FPR2	pEC_{50} (C43)
WT	8.10±1.13
110	ND
201	ND
205	ND
257	ND

c

FPR2	pEC_{50} (WKYMVm)	pEC_{50} (fMLFK)
WT	8.68±0.08	5.31±0.08
D106A	ND	ND
R201A	6.71±0.13	ND
R205A	ND	ND
W254A	ND	ND
V113A	ND	ND

	EC_{50} (nM) (fMLF)	EC_{50} (nM) (fMLFII)
wtFPR1	3.4±1.6	0.049±0.005
wtFPR2	2500±1200	6.9±1.3
FPR2 (D281G)	210±40	4.1±1.7

Supplementary Figure 6. Mutagenesis data on FPR1 and FPR2. (a) Expression levels of wild type FPR1 and FPR2 (WT) and mutants in HEK-293 cells determined by cell-surface staining using an Alexa647-labeled anti-FLAG antibody. Each data point was shown as mean ± SEM. n=3. **(b)** Calculated pEC_{50} values of different agonists in inducing signaling of different constructs of FPR1 and FPR2 based on the results shown in the main **Figure 3c**, **Figure 4a, Figure 5b,** and **Figure 6b**. WT represents wild type receptors. Each data point was calculated as mean ± S.D. n=3. **(c)** Upper pannel: pEC_{50} values of WKYMVm and fMLFK in inducing signalling of WT FPR2 and mutants measured by IP1 accumulation assays reported previously by Chen et al. [1]. Lower pannel: EC_{50} values of fMLF and fMLFII in inducing signalling of FPR1, FPR2, and the FPR2 mutant D281G measured by cAMP accumulation assays reported previously by He et al. [2]. ND (not determined) means undetectable signal in signalling assays.

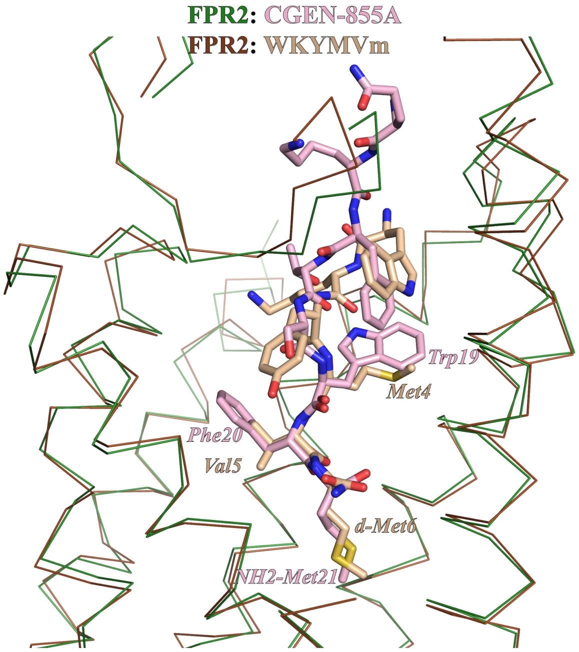

Supplementary Figure 7. Alignment of FPR2 bound to CGEN-885A and WKYMVm. The last three C-terminal residues in these two peptides are labeled.

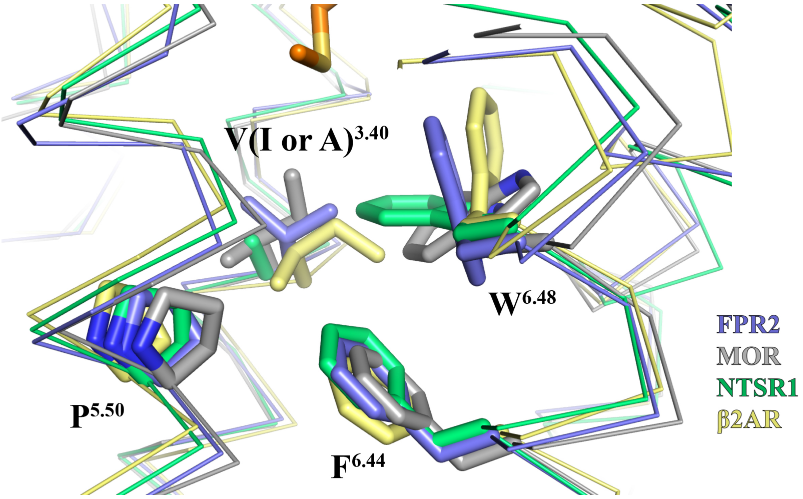

Supplementary Figure 8. Alignment of the four core residues in active GPCR structures. The active structures of FPR2 (with fMLFII), *μ*-opioid receptor (MOR, PDB ID 6DDE, http://doi.org/10.2210/pdb6dde/pdb), neurotensin receptor 1 (NTSR1, PDB ID 6OS9, http://doi.org/10.2210/pdb6os9/pdb), and β2-adrenergic receptor (β2AR, PDB ID 3SN6, http://doi.org/10.2210/pdb3sn6/pdb) are colored in slate, grey, light green, and pale yellow, respectively.

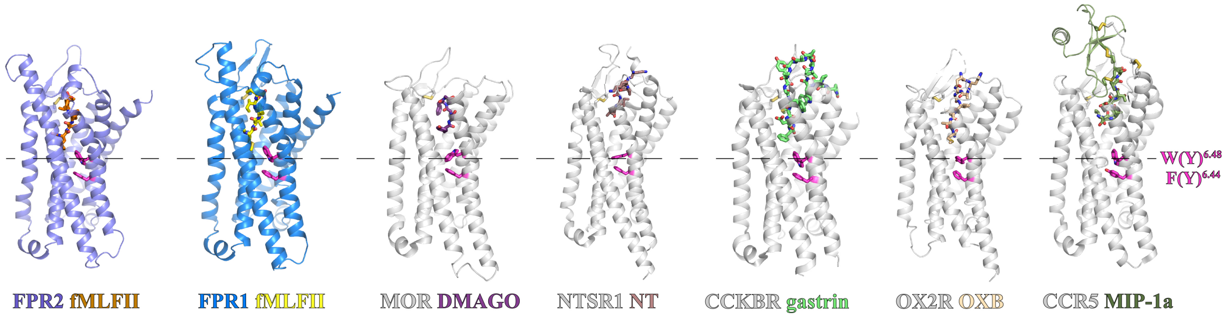

Supplementary Figure 9. Structural comparison of G_i-coupled Class A GPCRs bound to peptide agonists. FPR1 and FPR2 are colored in blue and slate, respectively. All other GPCRs including μ-opioid receptor (MOR, PDB ID 6DDE, http://doi.org/10.2210/pdb6dde/pdb), neurotensin receptor 1 (NTSR1, PDB ID 6OS9, http://doi.org/10.2210/pdb6os9/pdb), chemokine receptor 5 (CCR5, PDB ID 7F1Q, http://doi.org/10.2210/pdb7f1q/pdb), cholecystokinin receptor B (CCKBR, PDB ID 7F8V, http://doi.org/10.2210/pdb7f8v/pdb), and orexin receptor 2 (OX2R, PDB ID 7L1U, http://doi.org/10.2210/pdb7l1u/pdb) are colored in grey. fMLII in FPR1 and FPR2 are colored in yellow and orange, respectively. The peptide agonists in other GPCRs, DMAGO in MOR, neurotensin (NT) in NTSR1, gastrin in CCKBR, orexin B (OXB) in OX2R, and MIP-1a in CCR5, are colored in purple, sand, green, wheat, and smudge, respectively. The conserved core residues W(or Y)$^{6.48}$ and F(or Y)$^{6.44}$ are shown as magenta sticks.

Supplementary Table 1. Cryo-EM data collection, refinement, and statistics

	FPR2-G_i			FPR1-G_i
	**fMLFII**	**CGEN-855A**	**Compound 43 (C43)**	**fMLFII**
**Electron Microscopy Data Bank (EMDB) accession codes**	EMD-25729	EMD-25728	EMD-25726	EMD-25727
**Protein Data Bank (PDB) accession codes**	7T6V	7T6U	7T6S	7T6T
**Data collection and processing**				
**Magnification**	81,000	64,000	64,000	81,000
**Voltage (kV)**	300	300	300	300
**Electron exposure (e^{-}/Å^2)**	70	61.8	61.8	70
**Defocus range (µm)**	-0.5 ~ -3.0	-0.5 ~ -3.0	-0.5 ~ -3.0	-0.5 ~ -3.0
**Pixel size (Å)**	1.071	1.08	1.08	1.071
**Symmetry imposed**	C1	C1	C1	C1
**Initial particle projections (no.)**	5,430,289	3,057,898	4,082,666	2,002,737
**Final particle projections (no.)**	303,372	594,109	296,082	258,740
**Map resolution (Å) (FSC=0.143)**	3.1	2.9	3.0	3.2
**Local map resolution range (Å)**	2.4-5.0	2.4-5.0	2.4-5.0	2.4-5.0
**Refinement**				
**Initial model used (PDB code)**	6OMM	6OMM	6OMM	6OMM/6LW5
**Map sharpening *B* factor (Å^2)**	-110.70	-98.23	-107.73	-117.08
**Model composition**				
**Non-hydrogen atoms**	8951	8958	8907	8949
**Protein residues**	1142	1144	1136	1140
**Ligand**	1	1	1	1
**Lipids**	1	1	1	2
**R.m.s. deviations**				
**Bond lengths (Å)**	0.004	0.005	0.004	0.005
**Bond angles (°)**	0.620	0.627	0.601	0.680
**Validation**				
**MolProbity score**	1.42	1.54	1.58	1.81
**Clashscore**	7.60	8.54	10.69	14.48
**Rotamer outliers (%)**	0.00	0.00	0.00	0.00
**Ramachandran plot**				
**Favored (%)**	98.04	97.61	97.86	97.23
**Allowed (%)**	1.96	2.39	2.14	2.77
**Disallowed (%)**	0.0	0.0	0.0	0.0

Supplementary References

1 Chen, T. *et al.* Structural basis of ligand binding modes at the human formyl peptide receptor 2. *Nat Commun* **11**, 1208, doi:10.1038/s41467-020-15009-1 (2020).

2 He, H. Q., Troksa, E. L., Caltabiano, G., Pardo, L. & Ye, R. D. Structural determinants for the interaction of formyl peptide receptor 2 with peptide ligands. *J Biol Chem* **289**, 2295-2306, doi:10.1074/jbc.M113.509216 (2014).
